# Tailoring of Textural Properties of 3D Reduced Graphene Oxide Composite Monoliths by Using Highly Crosslinked Polymer Particles toward Improved CO_2_ Sorption

**DOI:** 10.1021/acsapm.2c01421

**Published:** 2022-11-10

**Authors:** Iranzu Barbarin, Nikolaos Politakos, Luis Serrano Cantador, Juan Antonio Cecilia, Oihane Sanz, Radmila Tomosvka

**Affiliations:** † POLYMAT and Department of Applied Chemistry, University of the Basque Country UPV/EHU, 20018Donostia-San Sebastián, Spain; ‡ Biopren Group, Inorganic Chemistry and Chemical Engineering Department, Nanochemistry University Institute (IUNAN), Universidad de Córdoba, 14014Córdoba, Spain; § Inorganic Chemistry, Crystallography and Mineralogy, University of Málaga, 29071Málaga, Spain; ∥ Department of Applied Chemistry, University of the Basque Country, 20018Donostia-San Sebastián, Spain; ⊥ Ikerbasque, Basque Foundation for Science, Maria Diaz de Haro 3, 48013Bilbao, Spain

**Keywords:** reduced graphene oxide, polymer composites, 3D porous monoliths, microporosity, mesoporosity, CO_2_ capture

## Abstract

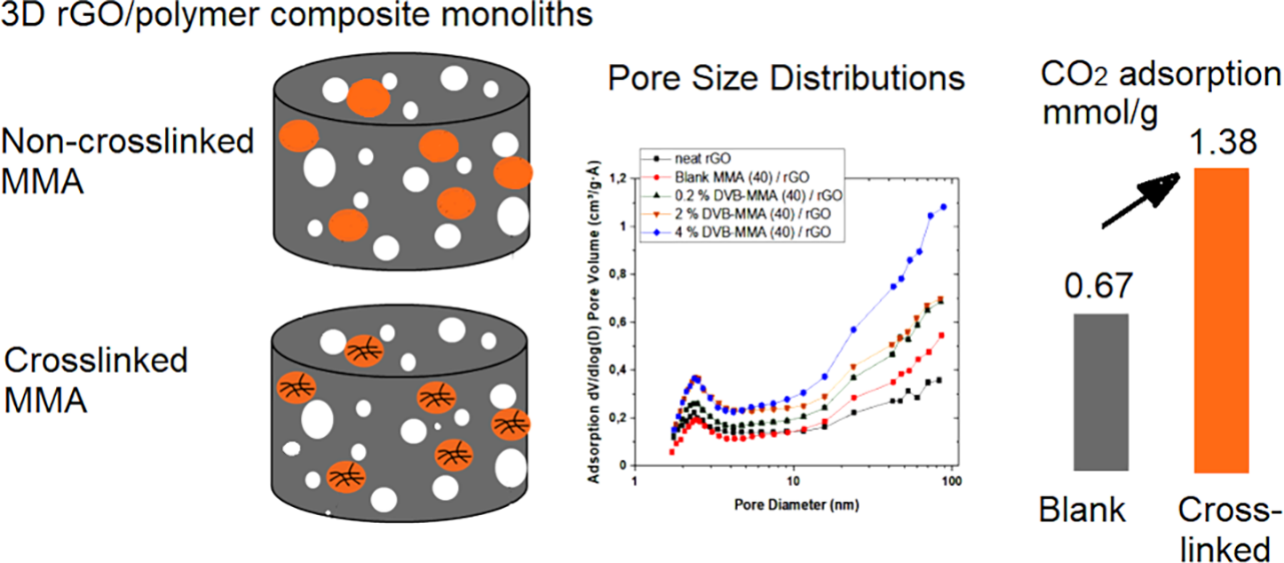

The main constraint on developing a full potential for CO_2_ adsorption of 3D composite monoliths made of reduced graphene oxide (rGO) and polymer materials is the lack of control of their textural properties, along with the diffusional limitation to the CO_2_ adsorption due to the pronounced polymers’ microporosity. In this work, the textural properties of the composites were altered by employing highly crosslinked polymer particles, synthesized by emulsion polymerization in aqueous media. For that aim, waterborne methyl methacrylate (MMA) particles were prepared, in which the crosslinking was induced by using different quantities of divinyl benzene (DVB). Afterward, these particles were combined with rGO platelets and subjected to the reduction-induced self-assembly process. The resulting 3D monolithic porous materials certainly presented improved textural properties, in which the porosity and BET surface area were increased up to 100% with respect to noncrosslinked composites. The crosslinked density of MMA polymer particles was a key parameter controlling the porous properties of the composites. Consequently, higher CO_2_ uptake than that of neat GO structures and composites made of noncrosslinked MMA polymer particles was attained. This work demonstrates that a proper control of the microstructure of the polymer particles and their facile introduction within rGO self-assembly 3D structures is a powerful tool to tailor the textural properties of the composites toward improved CO_2_ capture performance.

## Introduction

The anthropogenic CO_2_ quantity far overpasses the capacity of the natural processes to remove it, which results in a continuous increase in the annual growth rate of the CO_2_ atmospheric concentration. Among other effects, the accumulation of CO_2_ gas in the atmosphere promotes severe climate changes such as global warming, ocean acidification, and sea level rise.^1,2^ Electricity production by burning fossil fuels is one of the largest contributions to the anthropogenic CO_2_.^3,4^ However, technologies for environmentally friendly and economically viable large-scale electricity production are still in a phase of development.^5^ Carbon capture and sequestration (CCS) from postcombustion effluents plays a key role in alleviating ongoing emission levels and appears as a short-term solution or immediate action.^6^ According to a recent report on CCS, the global CO_2_ capture capacity has reached about 40 million tons by 2020.^7^ Nevertheless, to obtain a significant impact on climate change and to meet the Paris Agreement goal, the CO_2_ capture capacity should reach values of gigatons per year. This fact places the process of CCS on a critical position in the process of CO_2_ atmospheric concentration reduction.

The current capture technologies operate under significantly different conditions: precombustion capture processes usually operate at elevated pressures (about 30 bars) and a temperature of around 40 °C, while a typical postcombustion capture process from a coal-fired power plant operates close to atmospheric pressure and at 40–80 °C. This creates a requirement for a range of versatile adsorbents that have good stability and capacity for selective CO_2_ adsorption under different process conditions. Even though it has not or rarely been considered, a combination of various types of adsorbents could be a way to achieve this goal.

One of the perspective CCS technologies is based on carbon-based nanoporous materials. 3D graphene-based materials, a carbonaceous porous-type material, are seen as a spotlight due to their unique properties in terms of high surface area and porous texture and their versatility obtained through surface modification reactions that determines the surface chemistry and emphasizes adsorption performance.^8,9^ Nevertheless, the critical limitation of carbon-based adsorbents is the appropriate control over the pore size and pore size distribution, especially in the micropore- and small mesopore-sized regions, which may play a predominant role in CO_2_ adsorption.

On the other hand, the interest in porous organic polymers (POPs) as alternative CO_2_ adsorbents is growing rapidly due to their proper control of pore width and permanent porosity that make them promising materials for adsorption performance by the molecular sieve effect.^10^ The permanent porosity proceeds from wide chemical crosslinks between polymer chains that prevent their complete collapse, giving rise to a porous state.^11^ To date, a range of different POPs has emerged, such as polymers of intrinsic microporosity, covalent triazine frameworks, and hypercrosslinked porous polymers.^12,13^ For example, hypercrosslinked porous polymers are mainly prepared via Friedel–Crafts alkylation routes, resulting in surface areas of up to 2090 m^2^/g.^14^ Nevertheless, one of the disadvantages of microporous organic polymers is the diffusion limitation of the adsorbing gas due to the small pores. The diffusion will be very slow when the pore size is similar to the kinetic diameter of the CO_2_ molecule.^15^ In addition, in most of the cases, the synthetic approaches are environmentally unfriendly as high temperature, noble-metal catalysts, and the use of organic solvents are required.^16^

In this work, to take advantage of and to overcome the drawbacks of both 3D graphene-based structures and crosslinked polymers, hybrid structures made of 3D graphene-crosslinked polymer were synthesized. Due to the consistent mesoporous textural properties and high surface area, a functionalized surface rich in oxygen functional groups, and relatively high CO_2_ adsorption, the 3D graphene-based monolithic materials provide an excellent 3D adsorption platform. On the other hand, crosslinked polymers increase further the available surface area by providing micro- and mesoporosity induced by the stable covalent crosslinked structure, which will provide enhanced mechanical resistance,^17^ stability in cycle operations,^18^ and enhanced CO_2_/N_2_ selectivity.^19^

Moreover, to avoid high-energy-consuming processes and the use of volatile organic compounds in the synthesis of crosslinked polymers, in this work, polymerization in aqueous dispersed media was employed, producing polymer particles dispersed in water (latexes).^20,21^ These dispersions are shown to be a useful matrix for a self-assembly process of graphene oxide (GO) platelets after their reduction for the synthesis of composite monolithic 3D materials.^18,19^ The introduction of the particles provided excellent stability in cycle operations to the composites.^17,18^ Nevertheless, in most of the studied cases, the BET surface area of the composite materials dropped with respect to that of neat rGO, mostly because of the loss of the mesoporous structure. Subsequently, the CO_2_ capture performance was decreased for almost all composites with respect to the neat rGO monoliths. Under the conditions studied (25 °C and 1 atm), it was shown that the main characteristics affecting the CO_2_ adsorption are the BET surface area and the fraction of surface functionalization, while the microporosity did not show an important effect.^17−19^

To overcome the challenges while keeping the advantage of the polymer presence in the 3D structures, in the present work, a dense crosslinking was introduced within the waterborne polymer particles prior to their incorporation into the 3D materials. For that aim, methyl methacrylate (MMA)-crosslinked polymer particles with divinylbenzene (DVB) produced by free radical polymerization were employed. This synthesis technique offers production of large molar mass polymers and excellent control of the polymer microstructure and crosslinking density. The main idea is to increase the fraction of very small mesopores (<10 nm) to elevate the BET surface area.

For composite monolithic structure synthesis, GO dispersed in water was engaged as a precursor material and reduced under mild conditions after combination with polymer particles. In this way, the incorporation of crosslinked polymer particles to the graphene structures was straightforwardly attained. The influence of the polymer particles’ crosslinking density on the textural properties and CO_2_-philicity of the resulting hybrid monoliths was studied, with the main aim to introduce a control of the textural properties of the composite materials.

To the best of the author’s knowledge, this is the first time synthesizing graphene-based CO_2_ adsorption monolithic materials decorated with hard poly(methyl methacrylate)-crosslinked nanosized particles that synergistically contribute to the final characteristics and performance. While a reduced GO (rGO) 3D structure provided a CO_2_-philic large mesoporous surface rich in oxygen functional groups, the crosslinked polymer particles improved the thermal resistance of the material, adding microporous–mesoporous characteristics and improving further the CO_2_ capture capacity.

## Materials and Methods

### Materials

Graphene oxide aqueous dispersion (GO; 4 mg/mL) was used as supplied from Graphenea. It contains a monolayer content >95% and pH in a range of 2.2–2.5. The elemental analysis of the GO aqueous dispersion showed the following: C (49–56%), O (41–50%), S (2–3%), H (1–2%), and N (0–1%). Detailed characterization of GO dispersions is provided by the supplier in the following link: https://www.graphenea.com/products/graphene-oxide-4-mg-ml-water-dispersion-1000-ml.

l-Ascorbic acid (AsA, ≥99%, Sigma-Aldrich) was used as a chemical reducer. Technical monomers methyl methacrylate (MMA, Quimidroga) and divinylbenzene (DVB, 80% mixture of isomers, Alfa Aesar) were used without purification. Potassium persulfate (KPS, ≥99%, Sigma-Aldrich), sodium dodecyl sulfate (SDS, Sigma-Aldrich), and sodium bicarbonate (NaHCO_3_, Sigma-Aldrich) were used as received. In all polymerization reactions, deionized water was used. Tetrahydrofuran (GPC-grade THF, Scharlab) was used for the SEC instrument, and PS standards (Polymer Laboratories, VARIAN) were used for the calibration. 2-Propanol (HPLC grade, Sigma-Aldrich) and toluene (HPLC grade, Scharlab) were used as the internal standard and solvent in the GC technique, respectively.

### Synthesis of the Crosslinked Polymer Particles

Different degrees of crosslinked MMA polymer particles were synthesized by the seeded semibatch emulsion polymerization process. The DVB monomer was used as a crosslinker (0.2, 2, and 4 mol % with respect to MMA). The reactions were carried out in a glass reactor equipped with a N_2_ inlet, a reflux condenser, a thermocouple, a sampling tube, and a stainless steel anchor-type stirrer. The reaction temperature was managed by an automatic control system (Camille TG, Biotage).

The recipe used for the synthesis of the latexes is shown in Table 1 (for 0.2 mol % DVB). First, a seed with 20% solid content of MMA was synthesized by batch emulsion polymerization. A pre-emulsion was prepared by mixing MMA into the aqueous solution of surfactant (SDS) and buffer (NaHCO_3_) and loaded in the reactor, followed by addition of a water-soluble initiator (KPS). The reaction mixture was left to react for 2 h. Afterward, the monomer mixture (MMA and DVB), emulsifier (SDS), buffer (NaHCO_3_), and initiator (KPS) aqueous solution were fed for 3 h in two independent streams (F1 and F2, see Table 1). At the end of the feeding, the reaction mixture was allowed to react for 30 min batchwise to ensure complete monomer consumption. The reactions were carried out under a N_2_ atmosphere at 70 °C and under stirring at 200 rpm. With this procedure, latexes with a final solid content of 30% were prepared.

**Table 1:** Formulation Employed for the Synthesis of the Blank and 0.2 mol % DVB-Containing Latexes

material		seed (g)	MMA (g)	DVB (g)	SDS (g)	KPS (g)	NaHCO_3_ (g)	H_2_O (g)
seed			80		1.6	0.4	0.4	320
blank MMA	initial charge	20.8			0.085	0.078		32.1
	F1		117		1.43		0.65	205
	F2					0.275		11.625
0.2% DVB-MMA	initial charge	20.8			0.085	0.078		32.1
	F1		117	0.315	1.43		0.65	205
	F2					0.275		11.625

### Synthesis of 3D rGO/Polymer Hybrid Structures

The synthesis of the 3D rGO/polymer hybrid structures was based on a previously reported method.^18,19^ First, 40 mL of GO aqueous dispersion was sonicated at 25 °C for 1 h using a Hielscher Sonicator-UIS250v (amplitude of 70% and energy pulsed at 0.5 Hz, Hielscher Ultrasonics GmbH, Teltow, Germany). Then, the dispersion was stirred for 2.5 h at 80 °C.

The pretreated GO aqueous dispersion was mixed during 2 h with the aqueous dispersion of crosslinked polymer particles in quantities of 10 and 40% with respect to GO. Afterward, AsA reducing agent was added (GO:AsA mass ratio, 1:0.5) and stirred for 0.5 h. The sample was then placed in an oven at 90 °C overnight, which resulted in the formation of composite monolithic structures, in which all the solids placed in the initial dispersion (polymer and rGO) were incorporated. The monolithic hydrogels were purified by dialysis using deionized water. The conductivity of the residual water was measured until the value was lower than 10 μS/cm. Finally, the hydrogel was dried by a freeze-drying technique at −49 °C and 0.2 mbar using a Telstar LyoQuest 55 for 3 days.

### Characterization of the Crosslinked Polymer Particles

Monomer conversions and solid contents were determined gravimetrically. The average size of the polymer particles was determined using dynamic light scattering (DLS) in a Zetasizer Nano Z (Malvern Instruments). The gel content (GC %) of the polymer was measured by means of latex centrifugation in THF for 24 h under 4 °C and 15,000 rpm conditions (Sorvall Legend XTR, Thermo Scientific). The insoluble part was measured gravimetrically, and the gel content was calculated according to eq 1.
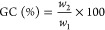
1where *w*_1_ is the amount of the total polymer added in THF, and *w*_2_ is the amount of the nonsoluble polymer that remained after centrifugation.

The molar masses of the soluble fractions obtained by centrifugation were measured using size exclusion chromatography (SEC) in THF at 35 °C with a THF flow rate of 1 mL/min. The SEC instrument consisted of an autosampler (Waters 717), a pump (LC-20A, Shimadzu), three columns in series (Styragel HR2, HR4, and HR6), and a differential refractometer detector (Waters 2410). The instrument was calibrated using the polystyrene standard, and the molar masses reported are related to polystyrene.

On the other hand, the crosslinking degree or crosslinking density of the polymer particles was related to the capacity of particle swelling in toluene (*g*_toluene_/*g*_polymer_). The strategy to analyze the swell capacity of the particles was the same as that described by Morton et al.^22^ For that aim, the latex was mixed with toluene (2 mL toluene/g of polymer) for 1 h, and then the mixture was centrifuged for 30 min at R.T. at 2000 rpm. Afterward, 2-propanol was added as the internal standard and the amount of polymer particles swollen in toluene was determined by a gas chromatograph (GC-14A, Shimadzu). The column employed for separation was a 50 m BP624 (from SGE Analytical Science), with an inner diameter of 0.53 mm and a film thickness of 3.0 μm. The calibration curve for toluene is presented in Figure S1 in the Supporting Information.

### 3D Graphene-Polymer Structure Characterization

The thermal stability and residual oxygen-containing functional groups of the 3D structures were measured by thermogravimetric analysis (TGA) in a TGA/DSC 3+ apparatus (Mettler Toledo). Two milligrams of samples was heated in a N_2_ atmosphere (50 mL/min) at 100 °C during 30 min, and then, the temperature was increased to 800 °C at a rate of 5 °C/min.

Scanning electron microscopy (SEM) was used to examine the porous structure using a Hitachi TM3030 tabletop model at an accelerating voltage of 15 kV after samples were coated with a thin gold layer.

Transmission electron microscopy (TEM) was used to observe the structures of the monoliths on the nanolevel using the Tecnai G2 20 Twin device at 200 kV (FEI Electron Microscopes). Before the analyses, the materials were embedded in epoxy resin, from which ultrathin sections (80 nm) were cut with a diamond knife on a Leica EMFC6 ultramicrotome device and placed on a 200 mesh copper grid.

The textural properties of the monoliths were characterized by means of N_2_ adsorption–desorption at −196 °C (Micromeritics ASAP2010 apparatus). Before the analysis, the samples were degassed at 60 °C during 8 h under vacuum. From N_2_ adsorption–desorption isotherms, the specific surface area (*S*_BET_) was estimated using the Brunauer–Emmett–Teller (BET) equation. Furthermore, the pore size distribution (PSD) was calculated using the Barrett–Joyner–Halenda (BJH) method, and the *t*-plot method was used for calculating the micropore volume (*V*_micro_).

Finally, the monoliths’ CO_2_ adsorption capacities were determined from their adsorption isotherms, measured using a Micromeritics ASAP 2020 Analyzer at 25 °C and up to 1 atm (i.e., volumetrically). Prior to the measurements, materials were outgassed at 60 °C and 10^–4^ mbar during 8 h.

## Results and Discussion

### Characteristics of Polymer Particles

To study the effect of the crosslinking degree of the polymer particles on the BET surface area and CO_2_ capture capacity of the final hybrid monoliths, four different dispersions were synthesized. MMA was copolymerized by the free radical emulsion polymerization technique with different molar fractions of the DVB crosslinker: 0 mol % (denoted as blank MMA), 0.2 mol % (0.2% DVB-MMA), 2 mol % (2% DVB-MMA), and 4 mol % (4% DVB-MMA). The final MMA conversion, particle size, fraction of polymer insoluble in THF solvent (gel content, GC %), molar mass of the soluble polymer fraction, and particle swelling in toluene of the different polymers are shown in Table 2.

**Table 2:** Characteristics of the Polymers

material	MMA conversion %	*z*-average particle size (nm)	GC %	sol *M*_w_(kg mol^–1^)	*Đ*	particle swelling
blank MMA	97.2	181	0	438	2.5	0.14
0.2% DVB-MMA	99.4	198	76	239	2.8	0.12
2% DVB-MMA	98.1	204	88	59	1.7	0.086
4% DVB-MMA	97.5	169	85	2	1.1	0.067

In Table 2, it can be seen that high MMA conversion was obtained in all the cases, resulting in polymer particle aqueous dispersions (latexes) with an average particle size in the range of 170–200 nm. There was no gel formed in the case of neat MMA polymerization (blank MMA), indicating the exclusive creation of linear MMA chains because of a lack of extractable hydrogens in MMA units but as well because the disproportionation is the predominant termination mechanism of growing MMA chains.^23^ By addition of DVB even in such a small quantity as 0.2 mol %, 76% of the polymer was insoluble, likely due to the crosslinking of the MMA chains induced by DVB. Moreover, by increasing the DVB fraction, the gel content was raised up to almost 90% in 2 and 4% DVB-MMA polymers. The molar masses of the polymer soluble part decreased with increasing gel fraction due to incorporation of higher molar mass chains into the gel. The swelling degree shows that by introducing DVB, the polymer absorbed less solvent, probably because the created crosslinks between the polymer chains decreased their mobility and more compact structures were formed. The further drop of the swelling degree with the increased DVB fraction in the polymer is an indication of the increased density of the crosslinked points. This fact perfectly explains that 2 and 4% DVB-MMA latexes have similar gel content, although the swelling degree is lower for higher DVB content, which indicates that the crosslinking degree is raised for higher amounts of DVB and that the distance between the crosslink points is lesser in 4% DVB-MMA.

**Figure 1:**
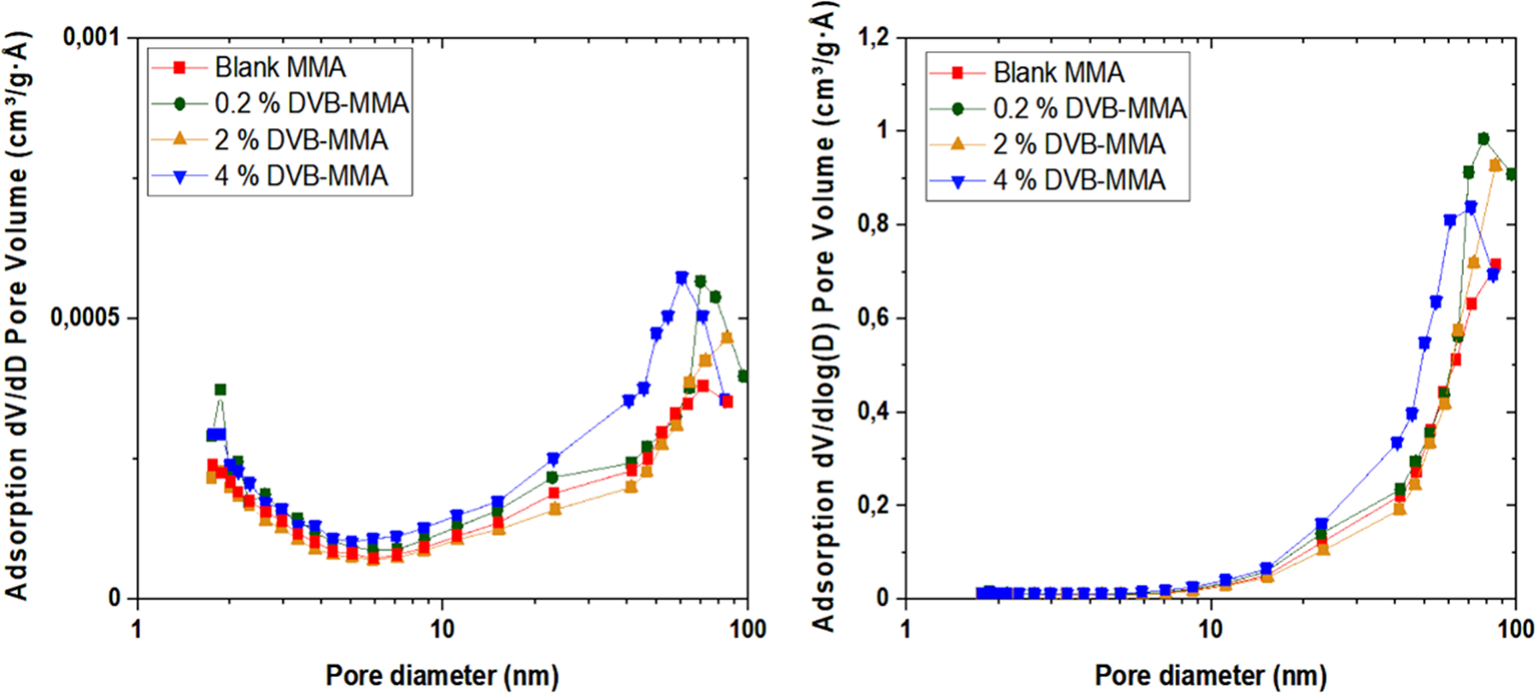
Pore size distributions d*V*/d*D* (left) and d*V*/d log *D* (right) of crosslinked polymer particles.

In Table 3, the textural properties (BET specific surface area (*S*_BET_, m^2^ g^–1^), total volume of the pores (*V*_total_, cm^3^ g^–1^), area of the micropores (*A*_micro_, m^2^ g^–1^), and volume of the micropores (*V*_micro_, cm^3^ g^–1^)) and CO_2_ adsorption (mmol g^–1^) of the crosslinked freeze-dried polymer particle are presented. In Figure S2 in the Supporting Information, the N_2_ adsorption–desorption isotherms are presented, and in Figure 1, the pore size distributions are shown for each polymer particle. In Figure 1, the left graph corresponds to the d*V*/d*D* curve, in which the contribution of smaller pores to the overall BET area is pronounced, while the right graphs correspond to d*V*/d log *D*, representing the contribution of the larger pores.^24^ These two ways of presentation were selected to get a complete image of how the crosslinking affects the distribution of both small and large pores.

**Table 3:** Textural Properties Determined from N_2_ Adsorption–Desorption Isotherms at −196 °C and CO_2_ Adsorption Capacities of the Polymer Particles at 25 °C and 1 atm

material	*S*_BET_(m^2^ g^–1^)	*V*_total_(cm^3^ g^–1^)	*A*_micro_(cm^2^ g^–1^)	*V*_micro_(cm^3^ g^–1^)	CO_2_ adsorption (mmol g^–1^)
blank MMA	34	0.343	<0.001	<0.001	0.30
0.2% DVB-MMA	41	0.442	0.860	0.001	0.31
2% DVB-MMA	33	0.385	3.264	0.001	0.31
4% DVB-MMA	42	0.385	5.793	0.002	0.32

MMA was copolymerized with DVB to control the textural properties in terms of the microporosity of the polymer particles that could improve the adsorption performance of the resulting monolithic materials. Table 3 shows that blank MMA with addition of 0% DVB did not present microporosity due to the more compact packing of the disorder and entanglements of the macromolecular chains. Instead, polymer particles containing DVB present higher surface area, amount of the total volume of the pores, area of micropores, and volume of micropores due to the developed pore structure. The pores are developed due to the decreased mobility and packing of the crosslinked chains. By increasing the DVB content, the crosslinked density increased too, as shown by the swelling degree (Table 2), due to the lower chain length between two crosslinked points, resulting in smaller pores and, consequently, more developed microporosity, as shown in Table 3. However, if the volume of micropores is compared with the total pore volume, it might be seen that it is negligible, which is rather strange if one takes into account the fact that the polymers containing DVB are more than 80% crosslinked. Likely, the mesoporous fraction was also increased with the crosslinking density, resulting in the similar contribution of the micropores to the porous structure.

To obtain deeper insight to this issue, pore size distributions were studied, as presented in Figure 1. It shows that the fraction of micropores and small mesopores ranging between 1.5 and 5 nm is similar in all the materials; however, the crosslinking introduced by addition of DVB contributed to the augmentation of the fraction of pores with an average diameter at 2 nm, especially for 0.2 and 4% DVB. The fractions of larger mesopores (20–50 nm) and macropores (50–100 nm) are more significantly different. By increasing the DVB content and crosslinking degree in the polymers, this fraction is larger and is shifted toward smaller size pores, likely due to the smaller distance between the crosslinked points. Taking into account the fact that MMA free radical emulsion polymerization is characterized by production of large molar masses,^25^ which according to Table 2 are about 400.000 Da (for blank MMA), apparently, the fractions of meso- and macropores were significantly raised.

**Figure 2:**
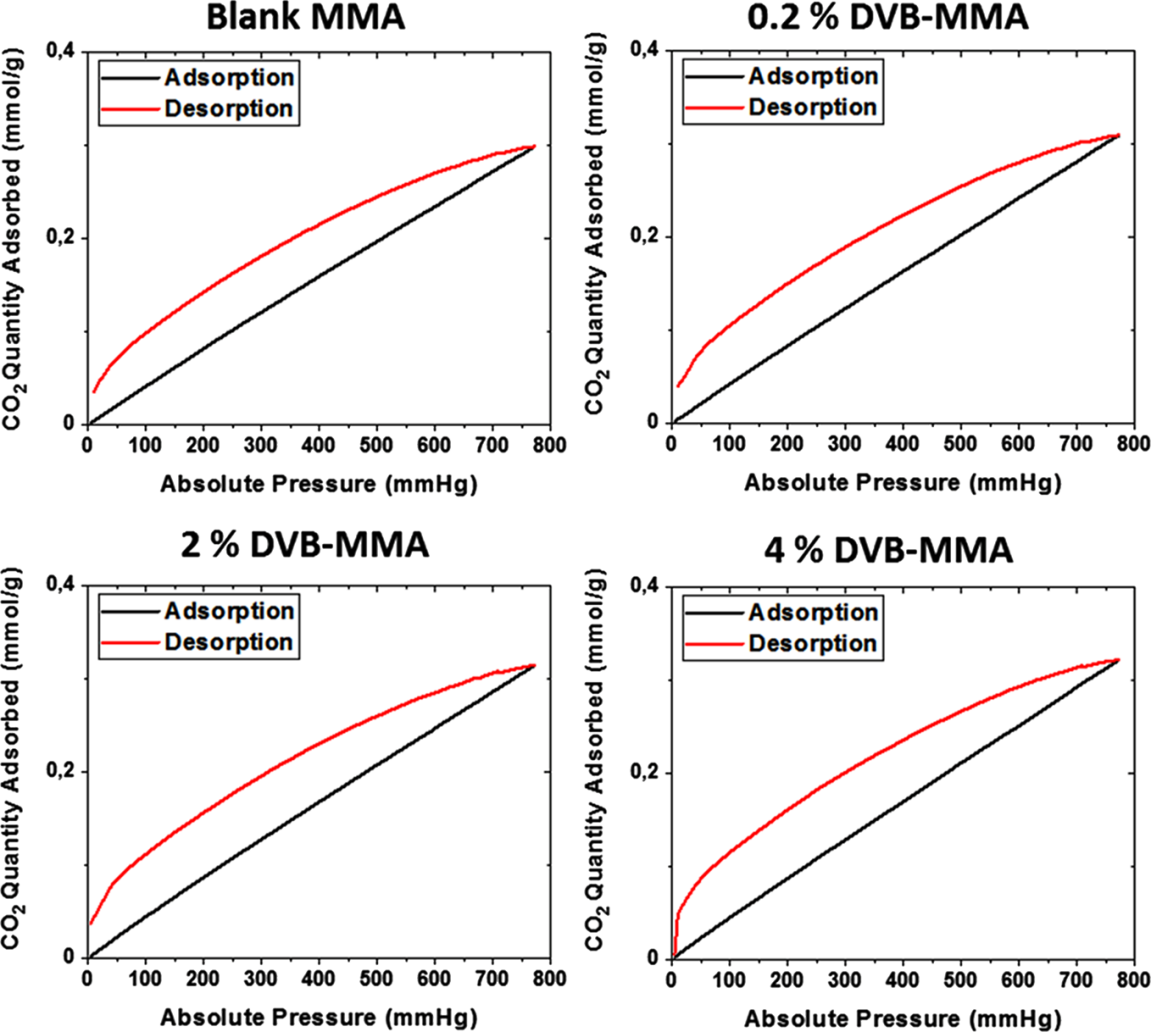
CO_2_ adsorption–desorption isotherms at 25 °C and 1 atm for different polymers.

The CO_2_ adsorption by these polymer particles is in the range of similar materials.^26^ The textural properties did not affect the adsorption capacity significantly, even though a slight increase with increasing porosity was observed. The CO_2_ adsorption–desorption isotherms obtained at 25 °C and up to 1 bar, as presented in Figure 2, show a linear increase in the absorbed CO_2_ quantity with pressure and a large hysteresis loop. The desorption process is much more energy-demanding, indicating more stable binding of the CO_2_ molecules, which need more energy to desorb, i.e., the gas is not released to the extent corresponding to the thermodynamic equilibrium value. Moreover, the textural properties of the materials may also have a contribution to this behavior, on the one hand, by the capillary condensation process occurring within the micro- and mesopores and, on the other hand, by the specific shape of these pores. According to the chemistry of the polymers, no chemisorption is expected to occur. On the other hand, as the same behavior is observed in the blank MMA polymer particles, it is a clear indication that the MMA chemistry is responsible for the possible stronger CO_2_ binding. It has been demonstrated theoretically that the ether and ester oxygen in the main polymer backbone or in the pending functionalities introduced strong CO_2_-philicity through specific binding that is sufficiently large to be important even at room temperature.^27^ Bonded in such a way, CO_2_ molecules would need higher energy for desorption than purely physisorbed CO_2_ molecules attached by van der Waals interactions, which can explain the large hysteresis observed in CO_2_ adsorption–desorption isotherms in Figure 2.

### Characteristics of 3D Hybrid Structures

To produce the hybrid monoliths, GO aqueous dispersion was mixed with an appropriate amount of polymer particle dispersions (10 and 40% polymer fractions based on the neat GO weight), during which process the polymer particles are adsorbed onto the GO platelets.^28^ Afterward, the chemical reduction eliminates the oxygen-containing functional groups of GO, and consequently, the hydrophobic character of the rGO platelets results in their self-assembly, attaining 3D rGO-polymer monolithic structures. For comparison, a neat rGO structure was produced too by reduction of pure GO aqueous dispersion without addition of polymer particles.

**Figure 3:**
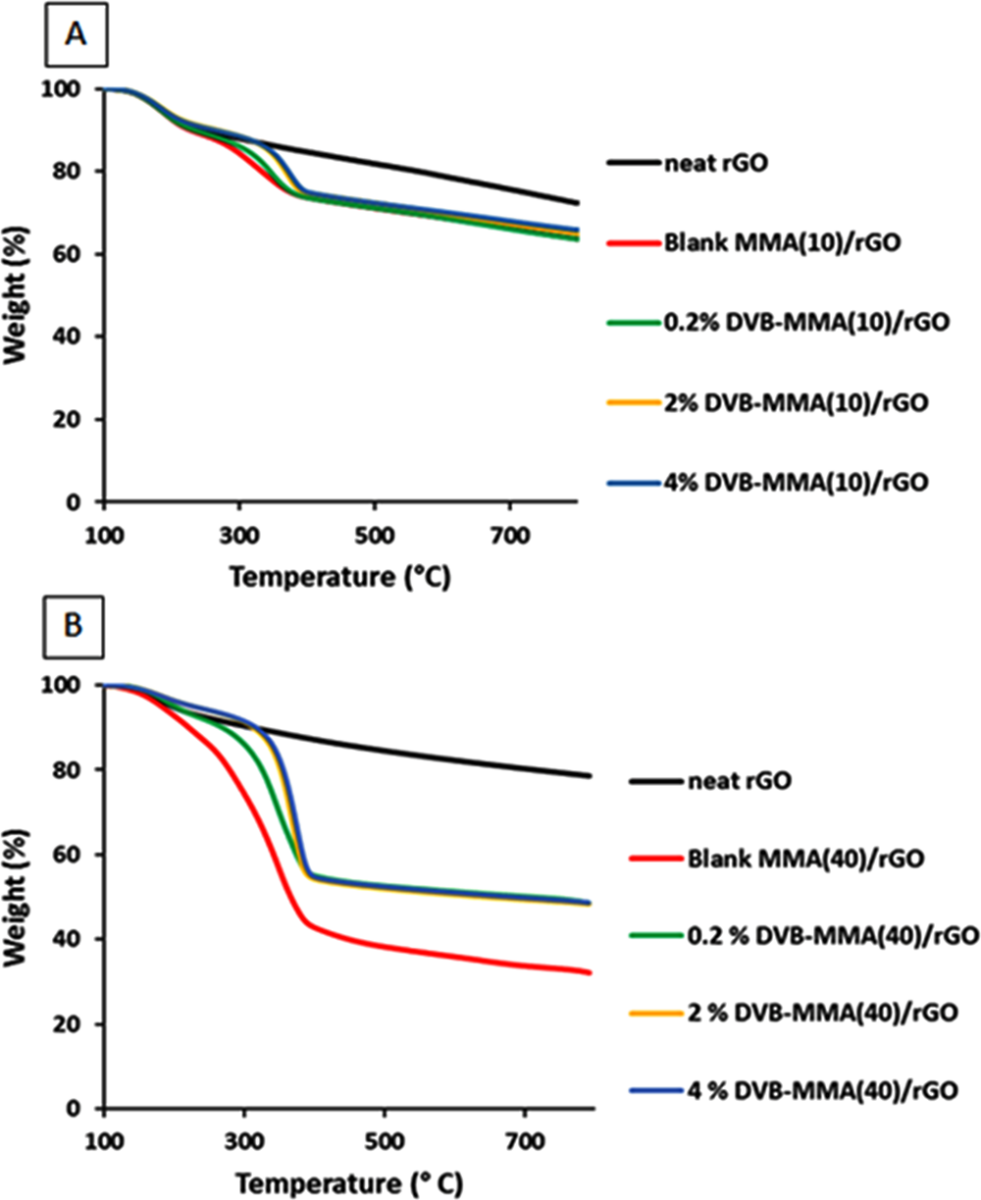
TGA thermographs of (A) monoliths with 10% polymer and (B) monoliths with 40% polymer. In both graphs, a neat rGO monolith was added for comparison.

Each of these hybrid materials was analyzed by TGA to study the thermal properties and to determine the residual amount of oxygen-containing functional groups. In Figure 3, the resulting thermographs are shown.

The first weight loss between 100 and 225 °C observed for all the materials, including neat rGO, corresponds to the residual oxygen-containing functional groups. The second weight loss region, between 300 and 400 °C, was attributed to the degradation of the polymer. The fraction of the residual oxygen groups for all materials is shown in Table 4. On the other hand, TGA curves revealed that the addition of polymer particles decreased the thermal stability, an effect that was lesser in the case of crosslinked particles. In fact, the monoliths with a higher crosslinking density (2 and 4% DVB-MMA/rGO with addition of both 10 and 40 wt % polymer particles) have a slightly improved thermal stability compared to the hybrid blank MMA/rGO structure. In both cases, the thermal degradation was postponed for about 100 °C when crosslinked MMA/DVB particles were added in the structure. This enhancement at a higher amount of DVB may be due to the aromatic nature of DVB that increases the onset temperature of degradation,^29^ but mostly this effect is due to the crosslinked structure of the polymer, which when more compact would need more energy for the thermal degradation.

**Table 4:** Fraction of the Residual Oxygen-Containing Functional Groups^a^

material	% O functionality	*S*_BET_(m^2^ g^–1^)	*V*_total_(cm^3^ g^–1^)	*A*_micro_(m^2^ g^–1^)	*V*_micro_(cm^3^ g^–1^)	micro (%)	CO_2_ adsorption (mmol g^–1^)
neat rGO	9.8	169	0.524	4.2	<0.001	0.2	0.87
blank MMA(10)/rGO	9.1	218	0.664	20.8	0.0059	0.9	1.06
0.2% DVB-MMA(10)/rGO	9.5	265	0.590	25.2	0.0083	1.4	1.08
2% DVB-MMA(10)/rGO	8.2	172	0.453	29.5	0.0093	2.1	0.96
4% DVB-MMA(10)/rGO	8.4	236	0.759	31.2	0.0091	1.2	1.17
blank MMA(40)/rGO	10.5	155	0.545	25.4	0.0092	1.7	0.67
0.2% DVB-MMA(40)/rGO	6.9	214	0.732	23.3	0.0057	0.8	0.84
2% DVB-MMA(40)/rGO	4.9	283	0.852	27.1	0.0080	0.9	1.38
4% DVB-MMA(40)/rGO	4.9	297	1.103	37.4	0.0107	1.0	1.01

a Textural properties of the 3D monolithic structures determined from the adsorption desorption isotherms at −196 °C and CO_2_ adsorption capacities determined at 25 °C and 1 atm.

**Figure 4:**
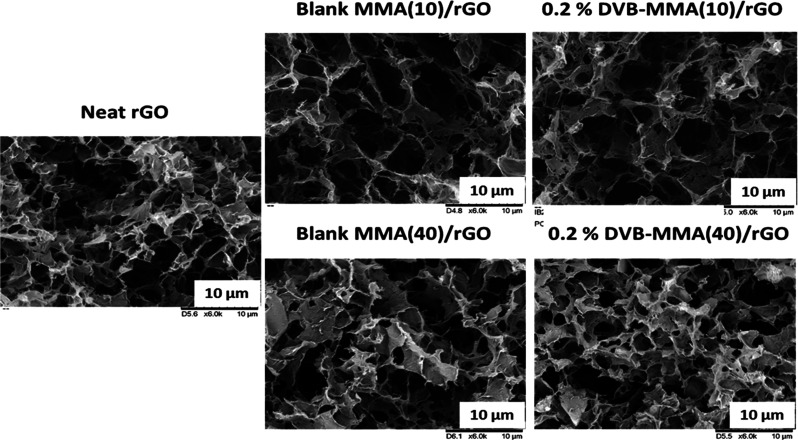
SEM images of monoliths containing 10 and 40% blank MMA and 10 and 40% crosslinked MMA polymers with 0.2% DVB and that of neat rGO (the scale bars in all images are 10 μm).

The morphology of the monolithic structures was characterized by SEM. In Figure 4, SEM images of monoliths containing 10 and 40% blank MMA and 10 and 40% crosslinked MMA polymer particles with 0.2% DVB and that of neat rGO are presented, whereas the monoliths with a higher quantity of DVB are shown in the Supporting Information, Figure S3. The SEM images reveal the highly porous morphology of all monoliths.

**Figure 5:**
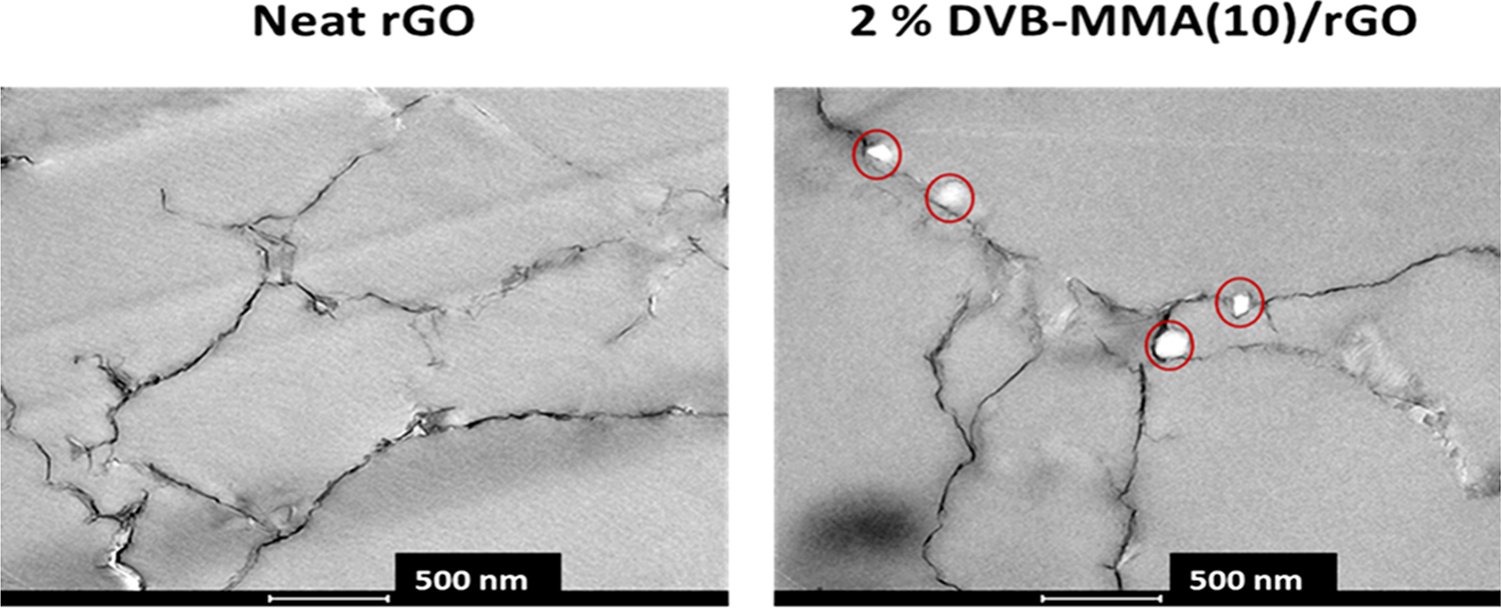
TEM images of neat rGO and 2% DVB-MMA(10)/rGO materials. Polymer particles are marked by red circles.

Figure 4 clearly shows that the morphological structure as observed in neat rGO was not affected by the presence of polymers in different quantities and with different crosslinking densities, presenting a very similar porous morphological skeleton. Furthermore, TEM analyses were performed, and the images obtained for neat rGO and 2% DVB-MMA(10)/rGO are shown in Figure 5.

In Figure 5, where TEM images of thin slices (80 nm) cut from monolithic structures embedded previously in the epoxy matrix are shown, the cross section of the rGO platelets can be clearly seen in both images as dark gray or black structures, whereas white polymer particles (marked by red circles) can be observed only in the composite monolith 2% DVB-MMA(10)/rGO. The size of the white structures matches the size of the polymer particles determined by DLS and presented in Table 2 (<200 nm).

**Figure 6:**
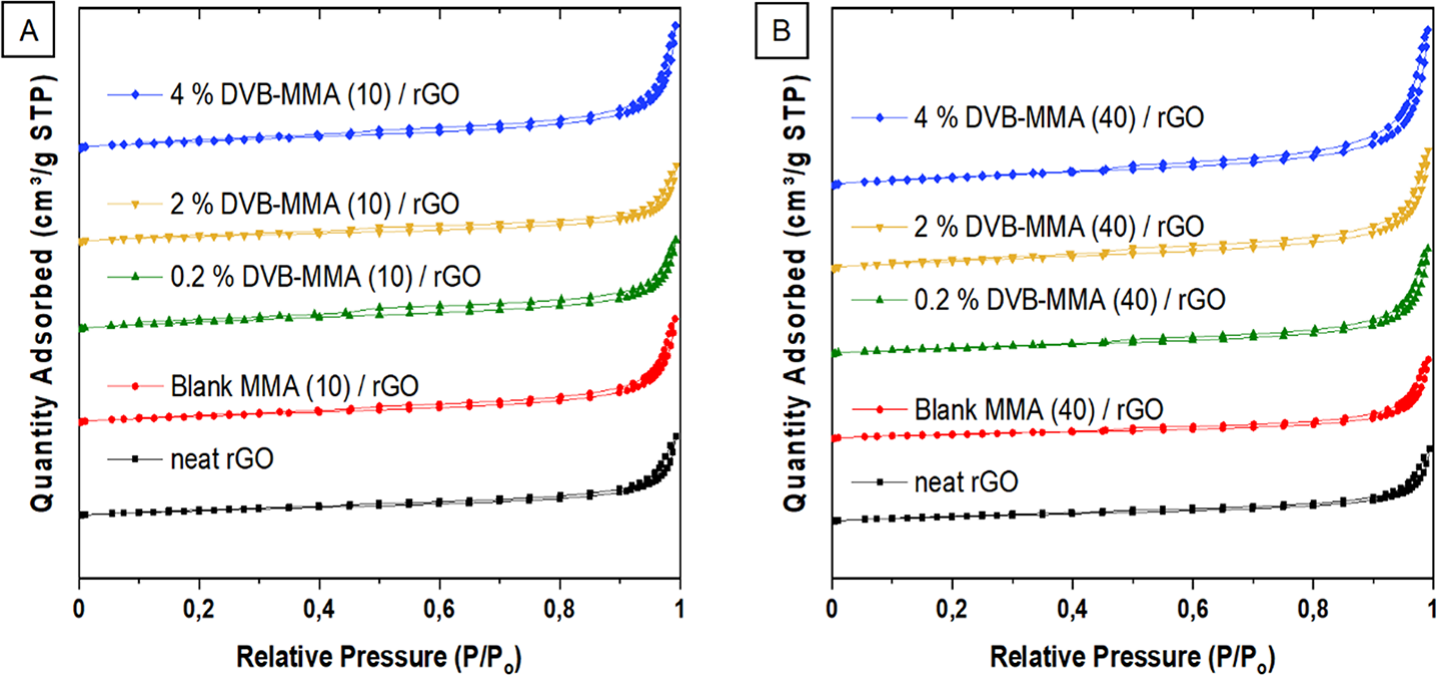
N_2_ adsorption–desorption isotherms for neat rGO and composites with the addition of 10 wt % polymer particles (A) and 40 wt % polymer particles (B).

**Figure 7:**
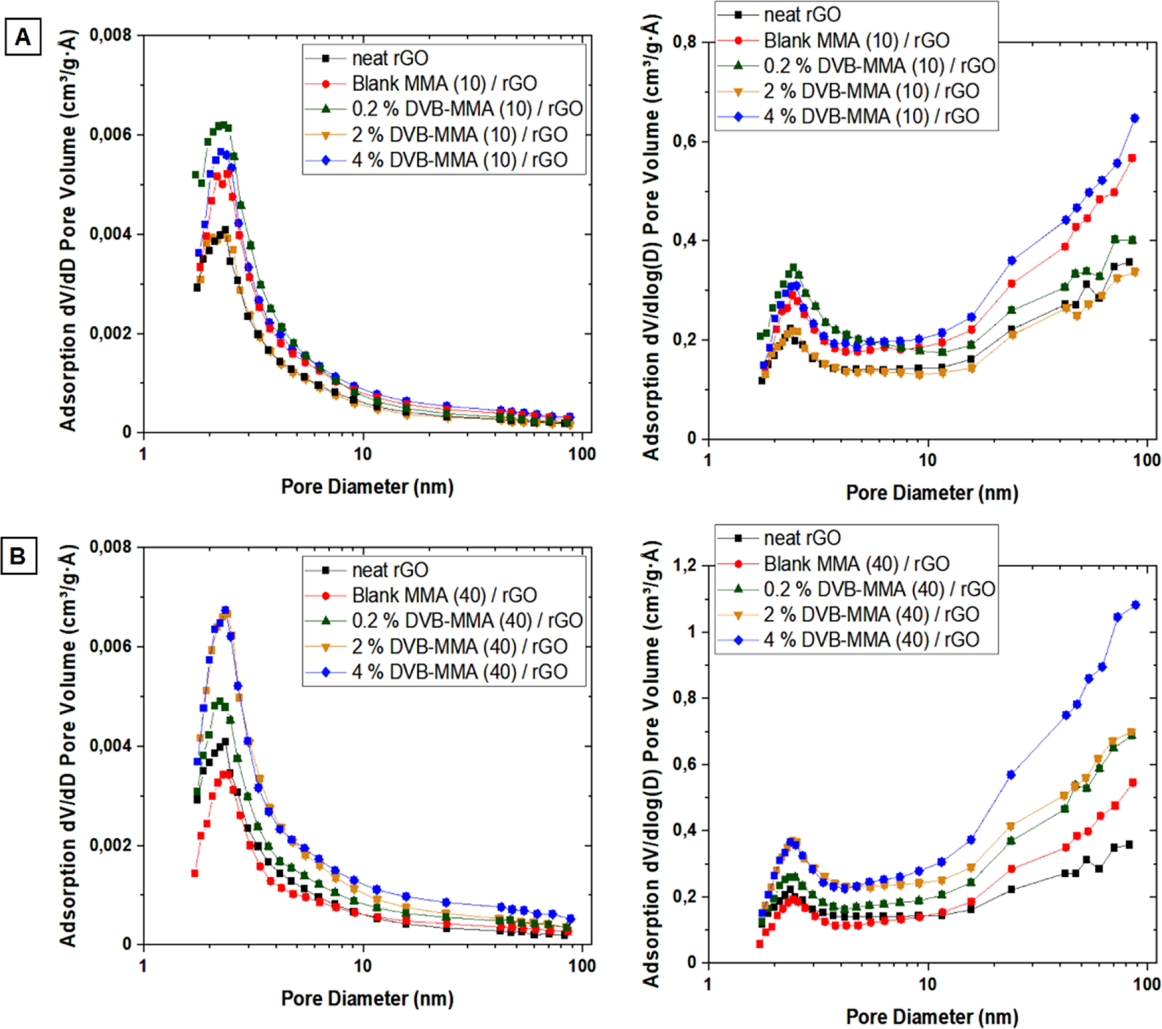
Pore size distributions d*V*/d*D* (left) and d*V*/d log *D* (right) of neat rGO and composite monoliths with the addition of 10 wt % polymer particles (A) and 40 wt % polymer particles (B).

To get deeper insight into the textural properties of the monolithic structures, the N_2_ adsorption–desorption isotherms are determined and presented in Figure 6. In Figure 7, the pore size distributions are shown.

According to Figure 6A,B, all the isotherms are of type IV, characteristic of mesoporous materials.^30^ The addition of either 10 or 40 wt % polymer did not alter the type of isotherm, which is in accordance with the SEM images (Figure 4 and Figure S3). In Figure 7A,B, the pore size distributions are shown, where it can be observed that in general, the composites have a higher volume of pores than the neat rGO monolith. On the one hand, the fraction of small mesopores in the range of 3–4 nm increased by addition of 10% blank MMA and crosslinked particles, likely due to the spacer effect of the particles that prevents the complete rGO platelet stacking during the self-assembly process, as observed previously.^19^ However, in the case of 40% polymer addition, the crosslinked particles induced further augmentation of the fraction of small and larger mesopores, as well as that of the macropores, an effect probably induced by the crosslinked structure of the particles.

In Table 4, the fraction of the residual oxygen-containing functional groups determined from the TGA curves, textural properties, and CO_2_ adsorption capacities are presented for neat rGO and the composite monoliths. The % micro in Table 4 corresponds to *V*_micro_/*V*_total_.

The fractions of oxygen functionalities within rGO, determined from TGA curves shown in Figure 3 as the fraction lost between 100 and 225 °C, were around 10% for 10% polymer added and about 7% for 40% with decreasing tendency in the case of crosslinked particles. Taking into account the fact that in the case of 40% polymer, fewer graphenic materials are present, even though the relative quantity of oxygen functionalities is lower, the functionalization level is similar.

The textural properties developed from the N_2_ adsorption–desorption isotherms at −196 °C are shown in Table 4. As predicted, higher BET specific surface areas were obtained within composite structures compared to the neat rGO, which is opposite to that obtained in all previous studies with noncrosslinked polymer particles.^17−19^ This is a consequence of the interplay between the following two effects. While polymer particles acted as spacers between individual rGO platelets, preventing their complete stacking during the self-assembly process and contributing to the formation of the microporous structure, their crosslinked morphology and internal porous structure contributed to the overall porosity of the composites. Considering the total volume of the pores, almost all composite monoliths presented higher porosity than the neat rGO structure. As mentioned, this is the first time attaining higher porosity by addition of polymer particles to the neat rGO structure, especially when 40% polymer was introduced within the structures.^18,19^ According to our previous works,^18,19^ functionalized noncrosslinked MMA polymer particle addition affected always negatively the textural properties and CO_2_ capture capacity. Therefore, the observed effect of increased porosity in this work is a clear effect of the crosslinked polymer chains within the particles. The fraction of micropores is higher for all composites than for the neat rGO; nevertheless, the effect is lesser in the case of 40% polymer. A probably much higher quantity of polymer particle spacers between the rGO platelets resulted in the formation of fewer micro- and mesopores. The particle diameter in the range of 170–200 nm between individual graphene sheets apparently created larger meso- and macropores.

**Figure 8:**
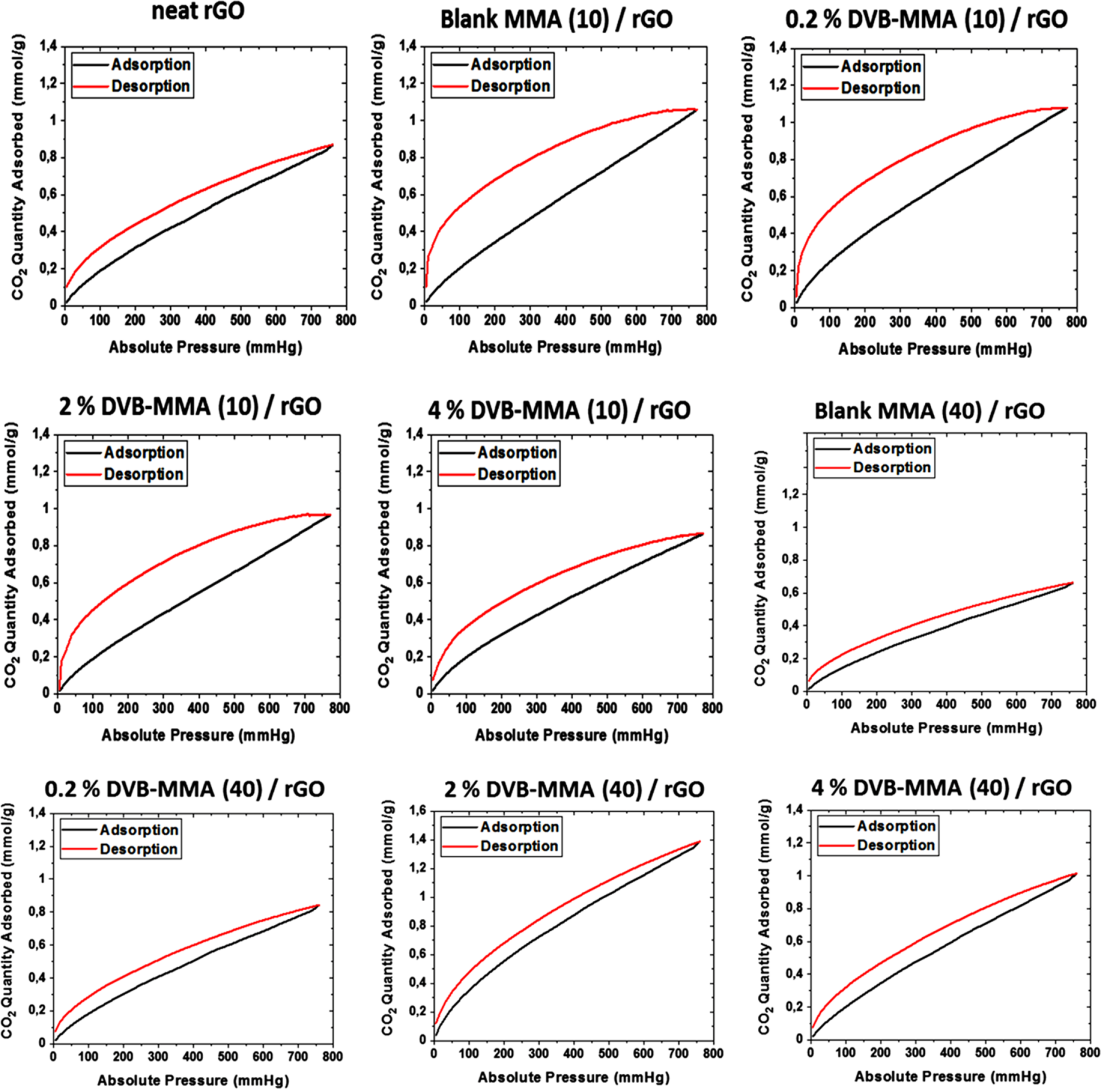
CO_2_ adsorption–desorption isotherms at 25 °C and 1 atm for 3D neat rGO and composite monolithic structures with different polymer quantities.

In Figure 8, the CO_2_ adsorption–desorption isotherms for 3D monolithic structures measured at 25 °C and up to 1 atm are presented. What is intriguing is that when 10% crosslinked particles are introduced within the structures, the CO_2_ adsorption–desorption behavior is similar to that of polymer particles shown in Figure 2, indicating that the polymer particles within the composite structure have a direct contact with CO_2_ molecules and affect the adsorption. Oppositely, when 40% particles are introduced into the composites, the CO_2_ adsorption–desorption behavior is similar to that of neat rGO material. Despite the much higher presence of polymer particles, probably they are included between the platelets that act as a barrier and hinder the direct contact between the polymer and CO_2_.

**Figure 9:**
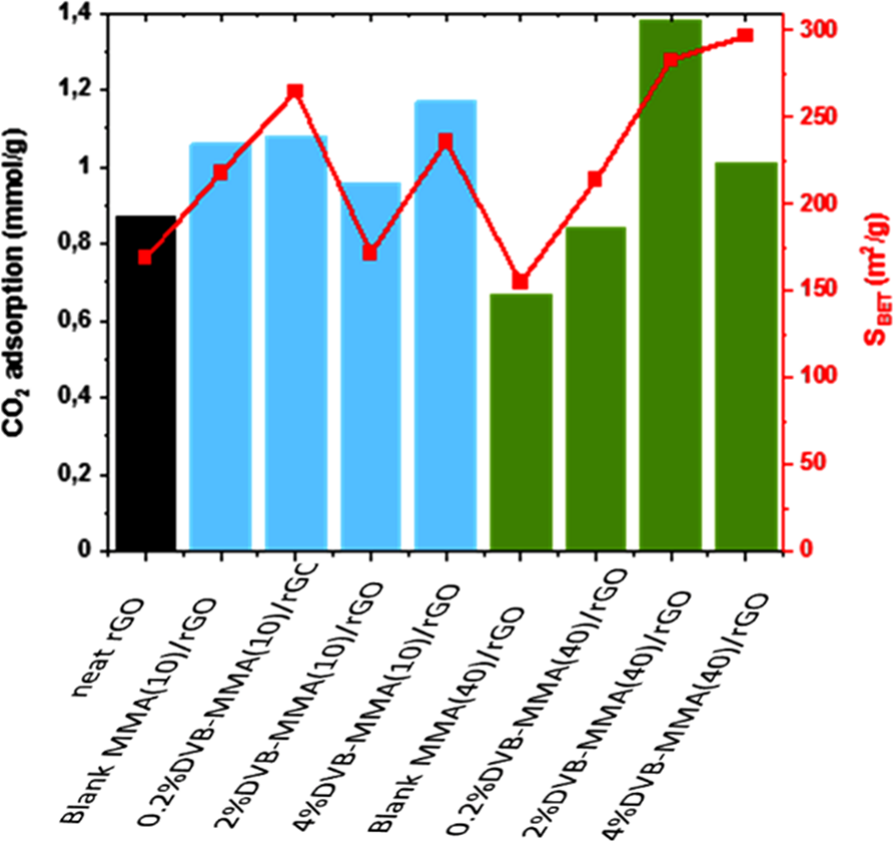
CO_2_ adsorption capacity and BET surface area of the different 3D monolithic structures. Black bars correspond to the neat rGO structure, blue bars to monoliths with the addition of 10% polymer particles, and green bars to monoliths with the addition of 40% polymer particles.

CO_2_ adsorption capacities of the monoliths are presented in Table 4 and Figure 9. When 10 wt % polymer was added, in all the cases, the CO_2_ adsorption improved with respect to the neat rGO monolith, obtaining the highest CO_2_ adsorption of 1.17 mmol/g, with the highest crosslinked polymer particle, 4% DVB-MMA(10)/rGO. The adsorption seems to be very similar, probably due to the similar chemistry and textural properties of the composites containing 10% polymer, and the introduced crosslinking does not have an important effect.

In contrast, when the added polymer fraction was 40 wt %, the CO_2_ adsorption performance increased with respect to neat rGO just in the case of higher amounts of DVB, whereas the crosslinking of the particles was favorable for the CO_2_ adsorption for all DVB quantities with respect to blank MMA(40)/rGO. The 2% DVB-MMA(40)/rGO and 4% DVB-MMA(40)/rGO structures presented 1.38 and 1.01 mmol/g CO_2_ uptake, respectively, which are the highest adsorption values achieved so far when so much polymer was introduced within the composites, according to our previous studies when noncrosslinked particles were used.^18,19^ Likely, the important augmentation of the BET surface area from about 155 m^2^/g for blank MMA(40)/rGO to almost 300 m^2^/g (the highest achieved in rGO/polymer composite monoliths) has the main decisive effect on the observed CO_2_ uptake rise. It is worth noting that the contribution of microporosity to BET is the lowest, which defines the 40% composites as highly mesoporous material and, as such, very favorable for CO_2_ capture under the studied conditions. Moreover, taking into consideration the fact that the quantity of rGO is less than 60% and that there is still decent fraction of oxygen functional groups, the graphene surface that, in majority, has the direct contact with CO_2_ is actually densely functionalized, which according to our previous experience is the most important parameter determining the CO_2_ adsorption capacity.

Therefore, the textural properties of the 3D composites can be improved further by increasing the contribution of all pore types, from micro- and mesopores up to macropores by changing the amount and type of crosslinked polymer particle. In this way, an excellent control of the microstructure of the graphene-based composite materials was achieved, a task that is still challenging when we speak about the carbonaceous porous absorbents.^31^ This meets the requirements and widens the application possibilities of these materials for the capture of CO_2_ under different conditions and implementation of this technology in different processes.

## Conclusions

The main aim of this work is to improve the control of the textural properties of 3D rGO/polymer composite monoliths and to increase their BET surface area toward enhanced CO_2_ adsorption. The approach was based on the synthesis of waterborne MMA-crosslinked polymer particles with different crosslinked densities to study the effect of the porous structure and the fraction of micro- and mesopores and how it affects the CO_2_ uptake. For that, four different particles were produced by emulsion copolymerization of MMA with different amounts of the crosslinker DVB, and they were added to the 3D rGO structures at two different amounts (10 and 40 wt %). The monoliths were synthesized by a simple mixing of the GO platelets and the polymer particles in aqueous dispersion, which after addition of the reducing agent and reduction temperature were self-assembled into composite monolithic porous structures.

The crosslinked polymer particles with a higher DVB amount presented denser crosslinking and shorter distance between the crosslinking points, resulting in an increase in the BET specific surface area, total volume of the pores, and area and volume of micro- and mesopores.

Besides augmentation of the fraction of micro- and mesopores in the structures by addition of 10 wt % particles, there was no important differences observed in the chemistry (oxygen functional group quantity on rGO) and BET surface area, resulting in rather similar affinity toward CO_2_ of around 1 mmol/g, independent of the microstructure of the polymer particles (crosslinked or not).

Nevertheless, in the case of 40% polymer particles, the textural properties were importantly affected, and the porosity was increased by augmentation of all pore types, resulting in a significant rise in the BET surface area. The rGO surface was more densely functionalized too, altogether causing an augmentation of the CO_2_ uptake.

Because of the proper control of the microstructure of the polymer particles, the characteristics of the 3D graphene-polymer monoliths can be tailored by a simple procedure, resulting in competitive CO_2_ adsorption capacities for practical application. The advantages of the crosslinked polymer particles synthesized by emulsion polymerization are in their synthetic routes, monomer diversity, scalable technology, and potential low cost.

## Supporting Information Available

Calibration curve of toluene determined by GC; N_2_ adsorption–desorption isotherms for different polymer particles (blank MMA and crosslinked MMA with 0.2, 2, and 4% DVB); SEM images of monoliths containing 10 and 40% crosslinked MMA polymers with 2 and 4% DVB (PDF)
