# Supplementary material for: Tailoring of Textural Properties of 3D Reduced Graphene Oxide Composite Monoliths by Using Highly Crosslinked Polymer Particles toward Improved CO2 Sorption
Source: ACS Appl Polym Mater. 2022 Nov 10;4(12):9065–75. doi: 10.1021/acsapm.2c01421 (PMC9748741; doi:10.1021/acsapm.2c01421)
Supplement: Supplementary file 1 — ap2c01421_si_001.pdf [file ap2c01421_si_001.pdf]

Supporting Information

Tailoring of textural properties of 3D reduced graphene oxide composite monoliths by using highly crosslinked polymer particles towards improved CO_2 sorption

Iranzu Barbarin[a], Nikolaos Politakos[a], Luis Serrano[b], Juan Antonio Cecilia[c], Oihane Sanz[d] and Radmila Tomosvka[a,e,*]

[a]POLYMAT and Department of Applied Chemistry, University of the Basque Country UPV/EHU, 20018 Donostia-San Sebastián, Spain.

[b]Biopren Group, Inorganic Chemistry and Chemical Engineering Department, Nanochemistry University Institute (IUNAN), Universidad de Córdoba, 14014 Córdoba, Spain.

[c]Inorganic Chemistry, Crystallography and Mineralogy, University of Málaga, 29071 Málaga, Spain

[d]Department of Applied Chemistry, University of the Basque Country, 20018 Donostia-San Sebastián, Spain.

[e]Ikerbasque, Basque Foundation for Science, Maria Diaz de Haro 3, 48013, Bilbao, Spain

*Corresponding author: radmila.tomovska@ehu.eus

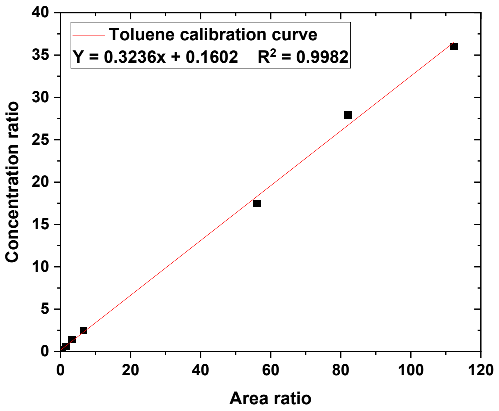

Figure S1: Calibration curve of toluene determined by GC.

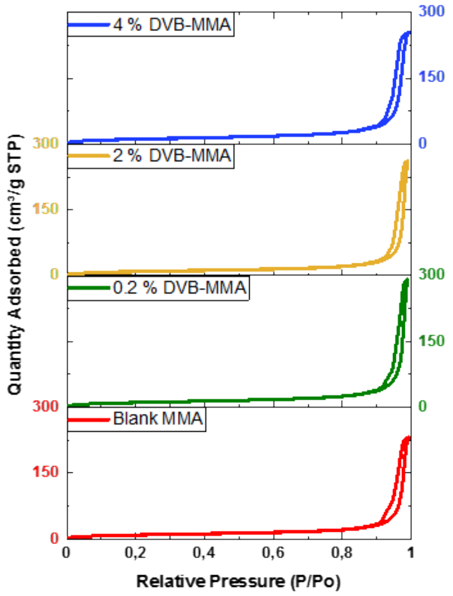

Figure S2: N_2 adsorption-desorption isotherms for different polymer particles (blank MMA, croslinked MMA with 0.2% DVB, 2% DVB and 4% DVB)

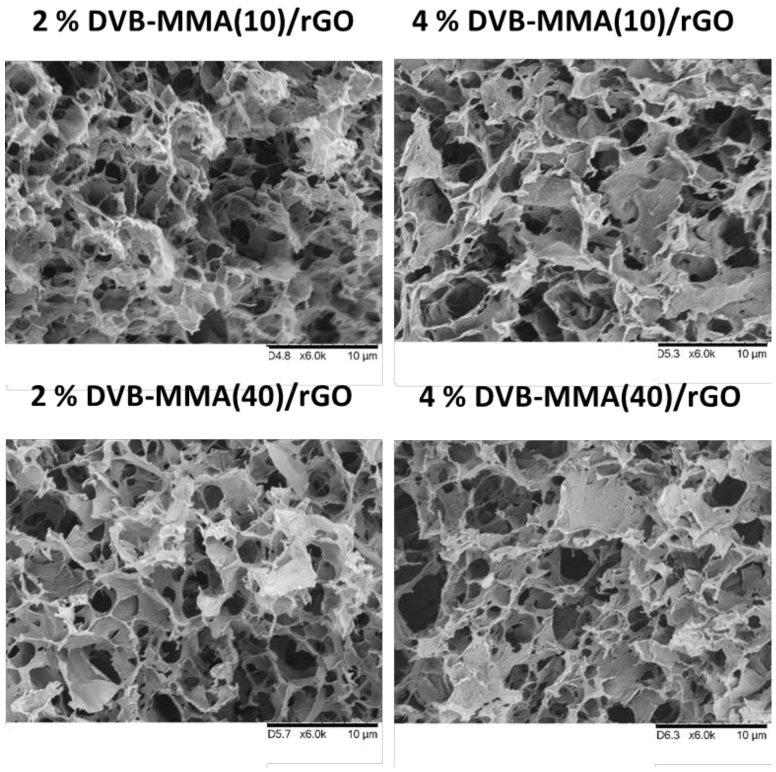

Figure S3: SEM images of monoliths containing 10% and 40% crosslinked MMA polymers with 2 and 4% DVB.
